# Digital Hydrologic Networks Supporting Applications Related to Spatially Referenced Regression Modeling^1^

**DOI:** 10.1111/j.1752-1688.2011.00578.x

**Published:** 2011-10

**Authors:** JW Brakebill, DM Wolock, SE Terziotti

**Keywords:** reach, network, modeling, streams, streamflow, SPARROW, NAWQA

## Abstract

**Abstract:**

Digital hydrologic networks depicting surface-water pathways and their associated drainage catchments provide a key component to hydrologic analysis and modeling. Collectively, they form common spatial units that can be used to frame the descriptions of aquatic and watershed processes. In addition, they provide the ability to simulate and route the movement of water and associated constituents throughout the landscape. Digital hydrologic networks have evolved from derivatives of mapping products to detailed, interconnected, spatially referenced networks of water pathways, drainage areas, and stream and watershed characteristics. These properties are important because they enhance the ability to spatially evaluate factors that affect the sources and transport of water-quality constituents at various scales. SPAtially Referenced Regressions On Watershed attributes (SPARROW), a process-based/statistical model, relies on a digital hydrologic network in order to establish relations between quantities of monitored contaminant flux, contaminant sources, and the associated physical characteristics affecting contaminant transport. Digital hydrologic networks modified from the River Reach File (RF1) and National Hydrography Dataset (NHD) geospatial datasets provided frameworks for SPARROW in six regions of the conterminous United States. In addition, characteristics of the modified RF1 were used to update estimates of mean-annual streamflow. This produced more current flow estimates for use in SPARROW modeling.

## Introduction

Processes controlling the supply, fate, and transport of chemical and organic constituents in terrestrial and aquatic systems occur throughout a watershed, from the headwater areas to the downstream receiving waters (Howarth *et al.*, 1996; Seitzinger *et al.*, 2002; Van Breemen *et al.*, 2002; McClain *et al.*, 2003; Alexander *et al.*, 2007). Spatially distributed physical characteristics are often used to describe such processes and conditions. These descriptions help formulate a better understanding of the intrinsic connections between land, water, and their interactions (Alexander *et al.*, 2007). A fundamental task prior to any quantitative approach to analyzing and validating these associations is defining logically connected spatial units that frame aquatic and watershed characterizations. Once developed, a consistent framework can be used to capture, store, and analyze relations and watershed characterizations within a geographically referenced network. Additionally, this information can be applied to hydrologic modeling applications designed to identify and investigate the spatial and temporal relations between the constituents and the processes that affect transport. Evaluating results of these modeling applications can contribute to the understanding of conditions and the management of activities related to the processes within the system (Kondolf *et al.*, 2002; Driscoll *et al.*, 2003; Grimm *et al.*, 2003; Alexander *et al.*, 2007; Gong *et al.*, 2009).

A digital hydrologic network of connected surface-water pathways and the areas they drain can be used as a foundation for a consistent spatial framework to characterize and analyze watershed processes. Discrete spatial units can help delineate, visualize, and spatially reference physical properties of a watershed system. These properties include landscape, aquatic and subsurface watershed characteristics such as contaminant supply, slope, soil characteristics, and annual streamflow. Hydrologic connectivity information from a digital network is equally important. The structure can facilitate the ability to simulate the movement of water and associated constituents within the system. This permits spatial analysis upstream and downstream relative to any location along a surface-water pathway. Additionally, information depicting the geographic connectivity provides the ability to establish and assess any spatial and temporal relations that may exist between the interactions of the associated watershed characteristics, the flow of water over the landscape, and within aquatic systems.

In this article, we provide a historical perspective of digital hydrologic networks and the origins of geospatial data that support the development and evolution of such networks. We also describe the important roles networks play in providing a spatial infrastructure for supporting hydrologic-transport models such as SPAtially Referenced Regressions On Watershed attributes (SPARROW). Modified versions of the River Reach File (RF1) (Horn *et al.*, 1994; USEPA, 1996) and the National Hydrography Dataset (NHD) (USGS, 1999b) are emphasized and discussed in the context of SPARROW model applications. These models have been developed in six regions of the conterminous United States and are presented in this issue. In addition, we describe an application that utilizes topological properties of a digital hydrologic network to estimate mean-annual streamflow in unmonitored stream reaches. This application produced more current flow estimates specified in the regional SPARROW models.

Using geographic information systems (GIS), spatially referencing various natural and human-related watershed characteristics to a digital network allows for the rapid display and analysis of the geographic distributions. Relative quantities and the factors related to supply, fate, and constituent transport can be evaluated in geographic detail. Spatially referenced point locations along the network where direct measurements have been collected over time (instream monitoring) provide the means to discern multiple relationships between watershed characteristics, processes, and the observations. These relationships can then be evaluated in both space and in time. Watershed characteristics and process descriptions and associations can also be utilized in a variety of applications to further determine and assess the connections and the processes controlling fate and transport. These applications include flux-based water-quality transport models and methods to estimate stream characteristics like flow and velocity in ungaged locations.

Many hydrologic models describe some aspect of the physical properties of the landscape. This includes the movement of mass in space and/or the change of mass in time. Models also can be designed to establish relations between water-quality monitoring, the supply of contaminants, and the natural attenuation processes that occur in transport across the landscape and within water pathways. Interpretations of model results can be useful at broad spatial and temporal scales to help address a variety of environmental-management decisions, including the design and tracking of contaminant reduction and protection strategies, monitoring practices and priorities, stream-health assessments, and regulatory requirements such as total maximum daily loads (TMDLs) (Schwarz *et al.*, 2006; Preston *et al.*, 2009). SPARROW is one such model that integrates monitoring and modeling. The watershed-modeling approach uses nonlinear statistical methods to define conceptual and spatial relations among quantities of contaminant sources, monitored contaminant flux, aquatic transport processes, and the physical characteristics that potentially affect contaminant transport to and within streams (Smith *et al.*, 1997; Preston *et al.*, 2009). A digital representation of a hydrologic network provides the fundamental framework for the spatial infrastructure supporting SPARROW models (Schwarz *et al.*, 2006). A linear network of stream reaches and associated drainage areas collectively form this basic foundation for spatially referencing monitored and predicted stream flux, quantities of potential contaminant sources, and stream and watershed characteristics to individual stream reaches and drainage areas (Schwarz *et al.*, 2006). This infrastructure also allows for a comprehensive, quantitative assessment of landscape characterizations and the relationships to water-quality conditions and the processes controlling supply and transport of constituents over a broad spatial domain rather than just at point locations where monitoring data are collected.

## Origins of Digital Hydrologic Networks

Many digital hydrologic networks used within the United States at regional scales have been derived from information collected by national mapping programs. The conterminous United States was mapped by topographic quadrangle at increasingly larger scales (i.e., finer detail) over a 100-year period until the early 1990s, when 1:24,000-scale mapping was completed (Kelmelis *et al.*, 2003). Mapping included, among other topics, the geographic locations of hydrologic features and interpreted elevation contours. The computer age facilitated a transition from maintaining and revising paper topographic maps to more efficient automated digital map-production procedures (Thompson, 1988). The National Digital Cartographic Data Base (NDCDB) was established by the U.S. Geological Survey (USGS) to distribute digital map data that adhere to map-production standards (McEwen and Jacknow, 1980). Digital line graph (DLG) files were one of the first national representations of cartographic hydrologic features in digital form available to the general public (USGS, 1989). DLG files are digital representations of selected cartographic data typically displayed on published USGS topographic quadrangle and sectional maps. The data structure of the DLGs maintained spatial relations of hydrologic features such as connectivity and adjacency between linear and areal features that allowed for simple plotting or analysis of their spatial relations (USGS, 1989). The files originated from small (1:2,000,000) to intermediate (1:100,000) scales, to large (1:24,000) scale topographic map series generated and revised over the last century. The NDCDB also contained digital elevation models (DEMs) derived from matrices of elevation points spaced at regular distances. Like the mapping programs, elevation points were compiled using various methods that have progressed over time and been produced at several spatial resolutions (USGS, 1987).

Linking geographic information to digital representations of hydrologic features is a logical progression from maps to GIS and subsequent frameworks supporting hydrologic modeling (Maidment, 1993; Brilly *et al.*, 1993). Early DLG files representing hydrology included an attempt to describe physical features of the mapped hydrography using a series of numerical codes. These codes were successful in identifying the cartographic features (like intermittent and perennial streams or rivers) once visualized on a paper map, but were limited in the ability to describe additional characteristics of the mapped feature. In addition, maintaining the hydrologic direction of flow of streams and rivers was subjectively determined at the time of data capture (USGS, 1989). Cartographic interpretations subject to human judgment in topographic map production (Thompson, 1988) and the physical condition (wetness of a particular year a survey was made) contributed to varying stream densities from quadrangle map to quadrangle map (Langbein, 1947). This inconsistency transferred to the digital products, thus challenging consistent regional assessments of hydrology.

In the early 1970s, the U.S. Environmental Protection Agency (USEPA) developed a hydrographic database of surface waters (RF1) for the conterminous United States (Horn *et al.*, 1994; USEPA, 1996). Designed to establish hydrologic ordering for navigation and modeling purposes, the river reach files progressed through numerous versions in an attempt to provide a common spatial framework that could be used to integrate numerous environmentally significant surface-water databases at reasonable spatial scales (Horn *et al.*, 1994; USEPA, 1996). The initial spatial framework consisted of digitized hydrologic features from National Oceanographic and Atmospheric Administration (NOAA) aeronautical charts at a 1:500,000 map scale (photo reduced from 1:250,000) and associated attributes contained in a single table. Representing the most detailed national hydrologic digital network of the time, RF1 introduced the concept of digital reach indexing for more than 60,000 streams nationally. Indexing allowed for a consistent framework that could take advantage of spatially referenced hydrologic information using a unique coding system and topological ordering of each stream reach for navigation and transport applications. Early applications included streamflow and velocity estimations, water-quality modeling, relocating point-source locations, and water-quality monitoring associations in support of the Clean Water Act (Horn *et al.*, 1994). However, some areas of the country exhibit unnatural differences in stream densities and spatial detail. In some instances, reaches were inappropriately connected at various locations, which could contribute to unexpected results. The physical locations of some surface-water pathways in RF1 are also suspect based on a comparison with landscape features such as elevation. Additional associated physical characteristics such as reach slope may be difficult to accurately calculate because of these discrepancies in location. In recent times, RF1 has been modified to address some of these deficiencies (see Supporting Information for details). In addition, catchments representing drainage areas for each mapped reach have been generated from various resolutions ranging from 30 m to 1 km cell sizes (Nolan *et al.*, 2002; Brakebill and Preston, 2003). Modified versions of RF1 and associated catchments have supported specific regional and national transport-modeling applications describing watershed conditions and are still widely used today (Horn *et al.*, 1994; Smith *et al.*, 1997; Alexander *et al.*, 1999; Preston and Brakebill, 1999; Nolan *et al.*, 2002; McMahon *et al.*, 2003; USEPA, 2007; Alexander *et al.*, 2008; Brakebill *et al.*, 2010).

The success of RF1 prompted the development of a more comprehensive hydrologic database. Reach File Version 3.0 (RF3) addressed the desire for more detailed hydrologic features at finer scales. Geographic representations of hydrologic features in RF3 were based on USGS 1:100,000 DLGs and provided a more detailed database for national, regional, and local reporting requirements such as those found in 305(b) sections of the Clean Water Act. Over 3,000,000 naturally flowing streams and constructed water bodies were represented nationally in RF3, a considerable increase from RF1 (Horn *et al.*, 1994).

RF3 and DLGs have since been incorporated into a NHD (Simley and Carswell, 2009). NHD maintains the ability to map hydrologic features in addition to an improved address system for spatial referencing and topological networking for stream navigation. NHD is currently being produced and distributed at medium (1:100,000-scale) and high (1:24,000-scale) resolutions (Simley and Carswell, 2009). NHD also supports systematic exchange, updates, and improvements to the data (USGS, 1999b). Specific protocols exist for updating features and related information. Stewards of the datasets are coordinated through partnerships among multiple federal, state, and local agencies. This coordination can potentially minimize the duplication of effort among stewards and provide a consistent, useful dataset for modeling and other water-resource applications.

A multi-agency effort has incorporated and expanded the capabilities of the medium-resolution NHD. NHDPlus (USEPA and USGS, 2005, 2009) is an application-ready product based on a 2005 snapshot of the medium-resolution NHD, the National Elevation Dataset (NED) (USGS, 1999a), and the National Watershed Boundary Dataset (WBD) (Simley and Carswell, 2009). NHDPlus can be used to improve the ability to study cause-and-effect relations in hydrologic processes and water quality at finer spatial scales (Alexander *et al.*, 2007; USEPA and USGS, 2005; Moore *et al.*, 2004). Regionally, NHDPlus provides significant spatial detail and a realistic representation of hydrologic pathways and numerous spatially referenced characteristics (attributes). Expanded capabilities of NHDPlus include updated reach-network connections and topology, 30 m elevation-derived catchments and flow paths (Johnston *et al.*, 2009), estimates of streamflow and velocity, and value-added attributes of spatially referenced landscape characteristics like land use and climate (USEPA and USGS, 2009). NHDPlus also integrates hydrologic features with other available data sources, including USGS streamgaging stations, the NED, and the WBD. The framework and subsequent tools developed for NHDPlus (and in development for high-resolution NHD) provide the ability to customize the behavior of the stream network, in addition to building and including user-defined attributes (USEPA and USGS, 2005, 2009). Because the locations of surface-water features within NHDPlus are based on cartographic interpretations, mapping anomalies of surface-water features also exist in the dataset. These include isolated (unconnected) stream reaches. Some isolated reaches are real features, but many are an artifact of the quadrangle map-production process, where in some cases, streams stop at the edge of quadrangle boundaries. These occurrences are currently being identified and corrected.

Associated NHDPlus attributes are stored in many external tables related to the surface-water features by common fields. The one-to-one and one-to-many relationships between geographic locations of surface-water features and other associated attributes can be complex compared to the simplicity of a dataset like RF1, in which all associated attribute information is stored in one table. This complexity is due in part to the fact that NHDPlus was designed to meet many purposes and utilizes several external data sources. In addition, NHDPlus contains a vast number of records. The ability to identify and connect to the proper table in which the information of interest is stored can be somewhat challenging (USEPA and USGS, 2009).

**FIGURE 1:**
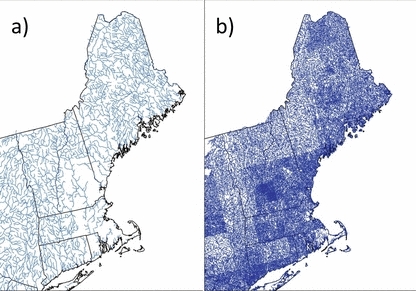
Maps Showing the Variations in Spatial Detail Within and Between (a) RF1 and (b) Medium Resolution NHDPlus in the New England Area of the Eastern United States.

Scale is important when determining stream networks for specific uses because scale will affect the number of streams defined (Alexander *et al.*, 2009). The number of streams, in turn, can affect the ability to determine the true catchment composition, stream length, and stream density. These are all useful quantitative measures of a stream network and the hydrologic responses of drainage-basin transport capacity and instream decay rates for contaminants in various stream sizes (Horton, 1945; Schwarz *et al.*, 2006). In addition, the availability and limitations of digital spatial data used to define, refine, and characterize hydrologic networks can vary in geographic extent, content, and scale, thus contributing to the complexity of regional assessments (Fargas *et al.*, 1997; Fekete *et al.*, 2001). Figure 1 demonstrates varying mapped stream densities between RF1 and medium-resolution NHDPlus in the New England area of the United States. When viewed from a regional perspective, inconsistencies between mapped quadrangles are evident in the NHD geospatial dataset. Inconsistent stream densities within the region are also evident in the RF1 dataset. These differences in stream densities are artifacts of scale and map-production techniques.

Continental- and global-scale elevation data, along with a variety of spatial analysis methods, have contributed to the ability to define and enhance hydrologic networks at various scales and geographic extents (Montgomery and Foufoula-Georgiou, 1993; USGS, 1997; Fekete *et al.*, 2001; Elliott *et al.*, 2005; Verstraeten, 2006; Davies and Bell, 2009). Basic applications of generating hydrologic networks from DEMs rely on the direction of surface-water flow from each elevation cell (flow direction) and the accumulation of cells flowing into any given cell (flow accumulation). Flow direction represents the steepest downslope direction that water on the land surface would flow. Once this direction is known, the number of cells flowing into any given cell can be calculated. In addition, surface-water flow pathways and associated catchment and watershed boundaries can be delineated (Jenson, 1991). One distinct advantage of DEM-based hydrographic analysis is that elevation-derived stream networks are not subject to potentially inconsistent cartographic interpretations. In addition, automated techniques used to generate stream networks and associated catchments can also be applied for repetitive procedures and outcomes. Such networks also are not limited by a fixed map scale like 1:100,000 or 1:500,000. However, they can be affected by inherent errors in the DEM creation process, causing potential misrepresentations and interpretations of the landscape, especially in areas of low relief. Networks also can be limited by the cell size (resolution) of the elevation data. Depending on the cell resolution of the DEM, hydrologic features can become oversimplified, contributing to a loss in accuracy or spatial detail. Ambiguities among the choices of search algorithms selected to create the network also can contribute to limitations and variations in interpretation. Consequently, stream networks and associated catchments derived from elevation data only may be inconsistent with previously accepted vector representations of hydrologic features (Saunders, 2000). Elevation-value adjustment is a potential remedy that addresses the misalignment of vector and DEM-derived networks to better align the hydrologic features with the topography of the landscape. This is the case when developing catchments for enhanced versions of the RF1 (see Supplementary Information for details) and the NHDPlus (Johnston *et al.*, 2009). The accuracy of the results can vary depending on the scale of the vector representations of surface-water features, cell resolution of the elevation data, and natural topographic relief of the area (Saunders, 2000; Wilson and Gallant, 2000). Elevation points collected at very close intervals, such as light detection and range (LIDAR) data, may be more representative of the topographic relief. These data can be used to produce detailed stream networks at very large scales. However, regional applications may be impractical using this type of data because of inconsistent data collection over large areas. In addition, extremely large computer files can be generated for even a small area, creating potential data-management issues and problematic results from network-generation algorithms.

## Digital Hydrologic Networks Supporting SPARROW

A digital representation of a hydrologic network is the fundamental framework of the spatial infrastructure supporting SPARROW models (Schwarz *et al.*, 2006). Collectively, the network of stream reaches and associated catchments form common spatial units used to frame aquatic and watershed characterizations. Associated reach characterizations and transport properties are then used by SPARROW to provide detailed spatial evaluations of the factors and processes affecting the source and transport of contaminants throughout the river network and its drainage area (Schwarz *et al.*, 2006).

The SPARROW model structure, in conjunction with spatially distributed characteristics within the hydrologic network, provides a statistical basis for empirically estimating stream-contaminant flux (predictions) in unmonitored areas. The SPARROW methodology allows for separate statistical estimates of spatially referenced explanatory watershed characteristics that quantify the amounts of contaminant sources individually or collectively. These estimates are weighted by established relations between constituent mass and other geographically referenced physical factors affecting aquatic and terrestrial contaminant supply, fate, and transport. Because of spatial referencing and network connectivity, estimates of contaminant flux can be quantified at any location along the network. These quantities of contaminant flux for each reach can be portrayed in specialized maps at multiple spatial scales to better interpret and visualize the contributions from individual contaminant sources.

**FIGURE 2:**
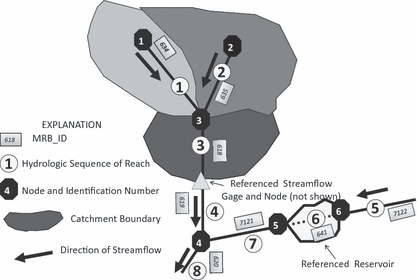
Schematic Drawing Showing the Spatial and Topological Relations Between a Reach, the Network Topology, a Monitoring Site, and Associated Catchments. Each reach is labeled with a unique identifier (MRB_ID) and a hydrologic sequence number (HYDSEQ). In addition, the terminal ends of each reach are labeled with node identifier (NODE-ID) values. The node at the downstream end of a reach is identified as the to-node (TNODE) and the node at the upstream end of a reach is identified as the from-node (FNODE).

A stream reach is the basic building block of the modeling framework (Figure 2). It is defined by a single vector representing surface-water pathways. Each reach extends either from headwater to stream junction, or from one stream junction to another stream junction (Brakebill and Preston, 2003; Schwarz *et al.*, 2006). Each reach is consistently oriented in the direction of streamflow and is usually connected to at least one other reach at its downstream node. Nodes are endpoints of lines that maintain the identity, direction, and location of intersected linear features. They are defined by a numbering system used to relate the upstream (FNODE) and downstream (TNODE) ends of connected stream segments (ESRI, 1992). This topological information is used to define reach-to-reach connectivity where the upstream node of a reach has the same identification number as the downstream node of the reach just upstream. Instances of a reach not connecting to another reach at the downstream node include: reaches that are part of a naturally closed basin; isolated reaches where the surface-water pathway is not clear and therefore not mapped; or a reach that is determined to be terminal, representing a subjective end to the stream (surface-water) transport.

Each reach also must be assigned a unique numerical sequence value (HYDSEQ) indicating its hydrologic order, from headwater to its termination point. Sequencing in a downstream direction based on reach-node topology gives an order to data processing that must be followed to route water from each stream segment to the next downstream segment throughout the network. This allows applications utilizing the network to accumulate mass referenced to the network in downstream order. Terminal reaches are defined as the last transport reach in which applications utilizing the networking capabilities terminate. SPARROW models compute the amount of flux prior to entering a fixed termination point such as a reservoir, international political boundary, or a terminal receiving body of water like an estuary or the ocean (Schwarz *et al.*, 2006).

While the stream-reach network describes the linear connection of surface-water pathways, the catchments defined by the area drained by each individual reach provide the ability to spatially reference land-based contaminant supply, load, transport, and load-prediction data. The catchments are typically derived from DEM data at a variety of resolutions ranging from 30 m to 1 km cell sizes, depending on the geographic scale of the application (Brakebill and Preston, 2003; Moore *et al.*, 2004; Alexander *et al.*, 2008; Hoos *et al.*, 2008;). Catchments are important to SPARROW models because they provide the spatial foundation to geographically reference explanatory parameters in manageable units and in spatial detail. In addition, delineated drainage catchments provide the ability to calculate required model parameters like local catchment drainage area and accumulated total upstream watershed drainage area. These parameters are used to normalize explanatory transport factors, quantities of contaminant supply, and loads predicted by the models. Additional details on catchment delineation supporting SPARROW modeling can be found in the Supporting Information of this article.

Independent of hydrologic sequencing, an additional numbering system unique to each individual modeling effort identifies each reach as a single linear spatial unit. This unique number is also shared by the catchment area drained by the reach, thus spatially linking the hydrologically connected streams and the respective drainage-area characteristics. This provides a numerical accounting system to aggregate spatially referenced GIS data layers to common spatial units. In addition, other relevant physical, environmental, and monitoring information can be associated to the common network and accessed using the unique identification number.

Most initial foundations of digital hydrologic networks supporting aquatic transport in SPARROW models are based on existing vector stream-reach networks such as RF1 and NHD. Enhancements to the datasets have been adopted in order to accommodate specific regional and national modeling applications. These enhancements include catchment generation for each stream reach, and topological corrections and attribute additions to the stream network such as stream monitoring, mean-annual flow estimates, and reservoir information (Smith *et al.*, 1997; Alexander *et al.*, 1999, 2008; Nolan *et al.*, 2002; Brakebill and Preston, 2003; McMahon *et al.*, 2003; Hoos *et al.*, 2008). The USGS National Water Quality Assessment (NAWQA) Program directed further enhancements to RF1 (MRB_E2RF1) in support of regional Major River Basin (MRB) SPARROW modeling presented in the SPARROW Featured Collection issue. These enhancements included the association of over 3,000 stream water-quality monitoring sites to stream reaches and updating estimated annual flows for each reach, later described in the application section of this article. Additional details related to these enhancements can be found in the Supporting Information of this article.

The NHD geospatial dataset was initially chosen to support nutrient SPARROW models in the Northeast region of the United States (MRB1) because RF1 in this part of the country contained inconsistent variations in stream density and lacked spatial detail (Moore *et al.*, 2004). However, in order to support regional SPARROW modeling applications, enhancements to NHD also were required. This included developing reach-node topology for stream routing and navigation, generating drainage catchments, and developing required model attributes like flow and velocity estimates and feature attributes such as land use. These requirements served as a catalyst for developing the NHDPlus dataset described earlier. Nationally, the NHDPlus dataset provides greater spatial detail for the locations of surface-water features, a larger number of stream reaches, and a smaller average catchment size than that of the modified RF1 dataset (Table 1). In the near term, the NAWQA Program intends to adopt NHDPlus as the foundation for future spatial networks supporting regional MRB SPARROW modeling. In addition, future snapshots of the NHDPlus are planned. Complexity is added because NHDPlus includes three ingredient databases (NHD, NED, and WBD) that continue to evolve and change. Each of these databases is maintained separately, but by tying NHDPlus updates into the maintenance structure of these core databases, SPARROW modelers can contribute improvements they make to the reach network. These updates could then be incorporated in future versions, as well as become available to the larger community.

**TABLE 1:** Comparision of the Spatial Resolution of Mapped Features in RF1 and NHDPlus. This table also compares estimates of mean annual flow and catchment size

Geospatial Dataset	Number of Catchments With Area	Number of Reaches With Mean Annual Flow Estimates	Number of Flowlines	Mean Catchment Size (km^2^)	Mean Annual Flow (m^3^/sec)	Median Flow (m^3^/sec^−1^)	Mean Reach Length (km)
RF1	66,147	63,018	64,696	129.7	68.9	2.0	16.0
NHDPlus	2,595,196	2,606,662	2,342,519	3.1	11.1	0.03	2.2

Notes: Mean annual flow for geospatial dataset RF1 represents original estimates. The number of flowlines represents the number of nonshoreline reaches.

A key network requirement for SPARROW modeling is the ability to connect pathways and associated characteristics hydrologically (Schwarz *et al.*, 2006). The spatial infrastructure of a SPARROW model developed partially or entirely from DEM-derived stream networks and flow paths across the landscape may provide more flexibility and spatial detail, thus furnishing a more precise framework to aggregate environmental explanatory and monitoring information at finer scales (Brakebill and Preston, 2003; Elliott *et al.*, 2005). The flow paths provide the hydrologic connection and essentially relieve the necessity for stream-node topology. This allows SPARROW to evaluate relations of sources and overland-transport properties to monitored streams at a grid-cell level as opposed to generalized or simplified catchment or watershed level, thereby enhancing the model's spatial detail. Elevation and flow-path information associated with the NHDPlus dataset currently provides this capability at a 30 m resolution (Schwarz *et al.*, 2006; USEPA and USGS, 2005; Johnston *et al.*, 2009).

### Spatial Referencing

A digital network managed within a GIS is essential to the SPARROW approach. In addition to model-required topological properties, the network provides a means to geographically locate stream-reach characteristics, independent (i.e., measured) response variables, explanatory variables, and predicted quantities of contaminant flux. Estimates of mean-annual streamflow and water velocity, reach length, mean-annual water travel time, reservoir surface area, and stream-reach type are examples of required characteristics referenced to the network and supplied to SPARROW models. Annual stream constituent-loading estimates serve as an independent response variable for SPARROW model calibration. Spatially referenced explanatory landscape, subsurface, and aquatic characteristics locate and identify quantities or surrogates of contaminant supply and the factors and processes associated with contaminant transport throughout the watershed. Geographically locating estimates of load (mass) on the reach network forms the basis to establish relations between constituent mass and the geographically referenced physical factors that may affect supply, fate, and transport (Smith *et al.*, 1997).

Mean-annual streamflow estimates are derived from preexisting estimates within the network datasets or reestimated for stream reaches using methods described in the applications section of this article. Each stream reach also is identified as either a reservoir or nonreservoir reach so that the appropriate aquatic loss rate can be estimated and applied to the modeled contaminant. Independent response variables used to calibrate SPARROW models are typically derived from station-specific empirical relations between individual water-quality concentrations, continuous stream-discharge data, and time. At each of the monitoring stations, which are geographically located on the appropriate stream reach, data are collected at a wide range of stream sizes by several state and federal agencies. Each unique monitoring-station identification number is maintained, allowing for additional station-specific analysis. Once associated with a stream reach, the connections within the reach network allow for systematic climbing upstream from each monitoring station, thereby associating the downstream measured load with every upstream reach until the next monitoring station is encountered. This ability to associate the monitoring station with upstream reaches ensures that the upstream sources of contaminants are accounted for in the observed load measured at the downstream monitoring location. Nodes can be placed at monitoring locations or at shorelines of impoundments for more accurate associations and assessments. Placing nodes at each monitoring location on a reach ensures that load estimates used for SPARROW model calibration are referenced to the downstream end of a reach, thus providing detailed spatial referencing. By adding a node at the monitoring location, the reach may be split (segmented) into two separate stream reaches, thereby creating a new reach upstream of the sampling site and maintaining the reach connectivity. In some cases, this approach may be desirable but impractical, and the monitoring site instead is associated with the closest downstream node of the reach (Figure 2).

The spatial arrangement and the variable characteristics identified within a hydrologic network can be important when evaluating spatial relations related to water quality and hydrology at regional scales (Verstraeten, 2006). Geospatial data layers representing these characteristics are critical to the evaluations using SPARROW. Geospatial data are merged with individual reach-catchment boundaries to spatially reference measures of contaminant supply and transport properties within a watershed. These quantitative values are then used by SPARROW as networked explanatory variables in the regression models. Contaminant sources can be viewed or evaluated in terms of their spatial distribution, relative supply contribution, and potential for transport to downstream waterways. Nutrient-source examples include point sources, land use, atmospheric deposition, and commercial fertilizer and manure applications (Schwarz *et al.*, 2006). Landscape and subsurface characteristics throughout the watershed also are merged with the catchments. These explanatory watershed characteristics are typically computed as mean values for each stream reach or normalized by catchment or watershed area. They represent physical properties and processes affecting the transport of contaminants as they move across the landscape and into streams. Examples include soil properties, slope, precipitation, and temperature (Schwarz *et al.*, 2006).

Geospatial data sources representing explanatory properties vary in detail, spatial extent, and scale. Data layers from local sources may be more spatially detailed and have more specific information content than sources developed at a regional or national scale. Land-use data, for example, exist in various media, temporal and physical scales, and classification schemes for many local communities. However, compiling a land-use dataset for a regional area using locally derived data would be impractical because of these variations. Agricultural census data are another example of a dataset with spatial variations in information content. Some relevant agricultural data are collected at the farm-field level but, for privacy reasons, are distributed to the public only as county-level information. Simply apportioning the same county explanatory data at finer resolutions based on aerial distributions within the network does not necessarily improve the quality of that information; however, methods do exist that allocate general explanatory information to finer-scale land-use data within a county and catchment (Schwarz *et al.*, 2006).

When developing a network intended to support SPARROW applications, one should consider the spatial extent, resolution, and the reasonable representations and variability of watershed characteristics, monitoring, and constituent-source data within the modeled area (Schwarz *et al.*, 2006). Information and detail are inherently generalized or simplified when explanatory data are spatially referenced to the hydrologic network. A network that is too coarse in scale relative to the explanatory information may negate, neglect, or oversimplify processes, sources, and effects on water quality. A network too detailed may not be able to distinguish any differences or detect any significant importance because of the differences in spatial detail between the network and the explanatory information. In addition, the constituent monitoring associated with the network also needs to be spatially detailed enough or representative of the varying conditions to detect variability in the explanatory information used in the model.

### Aquatic Transport and Decay

Streams, lakes, and reservoirs are known to trap sediment and contribute to nutrient losses (Chapra, 1997; Boyer *et al.*, 2006; Schwarz, 2008; Alexander *et al.*, 2009). Model-literature data suggest that physical properties of aquatic systems are related to nutrient loss rates and can be generalized over broad spatial scales (Boyer *et al.*, 2006). Because SPARROW statistically relates upstream contaminant sources to observed downstream loads, the explicit spatial structure defined by the stream portion of the network permits the simulation of material loss due to aquatic transport processes such as sedimentation and denitrification in streams, lakes, and reservoirs. These processes facilitate the accumulation or loss of associated contaminants and predicted quantities from each reach as they move downstream (Schwarz *et al.*, 2006) and are important components of contaminant mass balances in watersheds. Rates of instream loss or storage of constituent mass are computed in SPARROW as a function of streamflow and time of travel. Instream loss rates are estimated statistically and, for nonreservoir reaches, as a first-order decay rate for each stream class determined by the mean-annual streamflow estimate for each reach. Time-of-travel estimates are calculated as a ratio of reach length to mean stream-water velocity (Smith *et al.*, 1997; Seitzinger *et al.*, 2002; Boyer *et al.*, 2006). However, SPARROW models also permit stream channels to be considered a source of contaminant, such as in the case of phosphorus or suspended sediment (Schwarz, 2008; Brakebill *et al.*, 2010; Brown *et al.*, this issue).

SPARROW supports the designation of lakes and reservoirs spatially referenced to the network by locating impoundments on their corresponding stream reaches. Reaches are coded with an indicator, where 0 identifies a stream reach, 1 is an impoundment reach, and 2 is an outlet reach. The TNODE of the outlet reach type represents the impoundment location, and can be related to additional impoundment information such as surface area. Lakes and reservoirs also can be identified by determining the catchment in which they reside; this provides spatial referencing of impoundments that may be more detailed than the reach network. Aquatic loss or storage in reservoirs along the reach network is quantified in SPARROW as a settling velocity in units of length per time. The settling velocity is calculated as a function of the ratio of the outflow rate of the outlet reach (estimated mean-annual flow) and the surface area of the reservoir (Schwarz *et al.*, 2006). Lakes or reservoirs not on the reach network can be associated with the appropriate catchment and used as an overland land-to-stream transport factor, computed as the ratio of the number of impoundments to catchment area (Brakebill *et al.*, 2010). Major sources of reservoir information include Ruddy and Hitt (1990) and the National Inventory of Dams (USACOE, 2005).

### Predictions

**FIGURE 3:**
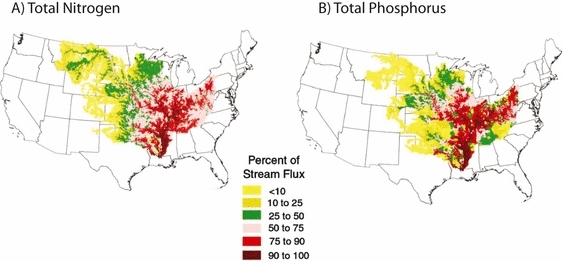
Map Showing the Percentage of Stream Nutrients Delivered to the Gulf of Mexico From the Incremental Drainage Based on SPARROW Predictions: (A) Total Nitrogen; (B) Total Phosphorus (from Alexander *et al.*, 2008, figure 3, supplemental information).

SPARROW can be used to predict flux for each modeled reach, including unmonitored locations. These estimates are a function of the established linear relations between monitored contaminant flux and the quantities of constituent supply referenced to the network (Schwarz *et al.*, 2006). Each stream reach and associated catchment is treated as an independent unit, quantifying the amount of contaminant mass generated within the catchment area and transported to the end of each stream reach. Contaminant mass that is generated locally for each stream reach is weighted by the amount of instream loss that would occur during aquatic transport. The cumulative loss of contaminant mass from its source through its continued transport downstream is dependent on the travel time and instream or reservoir loss rate of each individual reach. Mass-balance properties maintained by SPARROW provide a basis for flux accounting, whereby predicted flux can be allocated to its various upstream sources both geographically and by source type. Specialized maps make it possible to visualize contaminants discharged to estuaries attributed to specific sources from which they drain, providing guidance in managing the reduction of contaminant fluxes (Brakebill and Preston, 2004; Moore *et al.*, 2004; Alexander *et al.*, 2008) (Figure 3).

### Application

The ability to describe, route, and simulate the transport of constituents throughout the landscape is a key component to hydrologic analysis and modeling (Maidment, 1993). Flow characteristics of hydrologic networks are often the driving mechanism for modeling the transport of constituents such as nutrients and sediment. Unless flow characteristics can be determined reasonably well, the simulation of transport may be difficult (Moore *et al.*, 1991).

The RF1-based spatial framework (MRB_E2RF1) is well suited for enhanced attribution of features like annual streamflow critical to SPARROW models. The streamflow estimates originally associated with RF1 reaches were computed in 1982 by W.E. Gates and Associates, under contract with the USEPA (Horn *et al.*, 1994; USEPA, 1996). The methods are described in an unpublished report “Estimation of streamflows and the reach file.” Incorrect reach topology and inappropriate stream connections at various locations within the RF1 stream-reach network have been corrected when identified. Associated drainage catchments also have been created to reflect these changes. However, these deficiencies in the stream-reach network may have affected the original streamflow estimates with unexpected results. Therefore, the need for more accurate, current, and documented estimates led to the effort, described below, to develop a method to update streamflow estimates for each reach in the dataset that could subsequently be used in SPARROW modeling (Brown *et al.*, this issue). This methodology was designed such that it also could be used to improve the accuracy of the current estimated flows in more spatially detailed datasets like the NHDPlus.

In the application described here, long-term average-annual streamflow estimates for each of the approximately 60,000 reaches were computed for the period 1971–2000. The method incorporates the calculation of runoff (flow per unit area) for hydrologic cataloging units on the basis of historical flow data collected at USGS streamgaging stations, estimation of flow delivered to individual stream reaches from their local catchments, and the use of the enhanced reach network to accumulate streamflow downstream to terminal locations. A local bias correction in the flow estimation then is applied by calculating the difference between estimated and measured flow at streamgaging stations and then interpolating the bias throughout the river network. The entire process is repeated for each individual water year during the period 1971–2000. A water year is defined as the period from October 1 to September 30, and the water-year designation (e.g., 1971) corresponds to the year of the ending date (e.g., September 30, 1971).

### Computation of Hydrologic-Unit Runoff

Estimates of hydrologic-unit runoff were generated by combining historical flow data collected at USGS streamgaging stations, the respective drainage-basin boundaries of the streamgaging stations, and the boundaries of the 2,110 hydrologic units. Streamgaging stations for the analysis were selected for each water year based on the availability of a complete daily flow dataset for the water year. Geospatial data representing drainage-basin divides from the location of each streamgaging station were delineated using the NHDPlus dataset and the accompanying digital-elevation-model-based flow-direction information (USEPA and USGS, 2005). Basin boundaries with a computed drainage area within 25% of the streamgage drainage-basin area reported in the USGS National Water Information System (NWIS) (USGS, 2008) were considered valid for this analysis. In a typical water year during the period 1971–2000, there were about 6,000 streamgaging stations with a complete daily flow dataset and an acceptable drainage-basin boundary. The drainage-basin areas of these streamgaging stations ranged from 10 to 180,000 km^2^ with a median value of 3,000 km^2^.

Hydrologic-unit subbasins and their associated eight-digit accounting numbers (HU-8s) are a widely used geographic framework for the conterminous United States. Each unit defines a geographic area representing part or all of a surface-drainage area or a combination of drainage areas. Subbasins subdivide larger accounting units (HU-6s), subregions (HU-4s) and regions (HU-2s) into smaller areas designated by the U.S. Water Resources Council and the USGS's National Water Data Network. Subbasins range in size from 24 to 22,808 km^2^ with a median value of 3,133 km^2^ (Seaber *et al.*, 1987; Steeves and Nebert, 1994).

**FIGURE 4:**
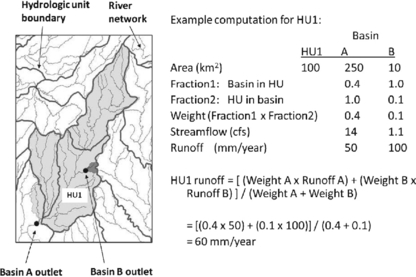
Runoff Computation for a Hypothetical Hydrologic Unit. Two basins are shown as filled polygons: basin A is light gray and basin B, which is nested in basin A, is dark gray. Hydrologic cataloging unit boundaries (HU-8s) are indicated by bold gray lines and one hydrologic unit is labeled HU1. Streams and streamgage locations are shown as thin gray lines and black dots, respectively.

Figure 4 illustrates the method used to compute runoff estimates for HU-8s. The first step is to compute runoff values (flow per unit area) for each streamgage basin by dividing the average daily flow for the water year by the delineated basin area. In a hypothetical example (Figure 4), runoff is estimated at two streamgaging stations (labeled A and B in Figure 4) by dividing the average daily flow measured at each of two streamgaging stations by their respective drainage-basin areas. (The drainage area of basin A is shaded light gray and the drainage area of basin B is shaded dark gray. Note that drainage basin B is nested within drainage basin A.)

Each geospatial basin boundary is then overlain on a geospatial dataset of HU-8s (the polygons outlined in bold black lines) to determine the area of intersection within the two datasets. For each overlapping area of HU-8s and drainage-basin boundaries, the fraction of the basin in the HU-8 and the fraction of the HU-8 in the basin are calculated. These fractions are then multiplied by each other to compute a weighting factor for each basin. The runoff values and associated weighting factors for all basins with any overlapping area with a HU-8 are combined, and a single weighted-average runoff value is computed for the HU-8 (Figure 4).

The weighted-average runoff computations illustrated in Figure 4 were repeated for all combinations of the roughly 6,000 basins and 2,100 hydrologic cataloging units (HU-8s). Runoff values for HU-8s that had no overlapping areas with streamgage basins were computed as the mean of the HU-8 runoff values within the same HU-4 (subregional unit).

### Intersection of HU-8 Runoff With Reach Catchments

The amount of flow delivered to each reach from its uniquely identified catchment was computed by intersecting the HU-8 runoff grid with MRB_E2RF1 catchments. Runoff values for each grid cell were summed based on the spatial intersection of the two datasets. The sum of grid-cell runoff values within each catchment has units of runoff [L·T^−1^] multiplied by area [L^2^] and can be expressed in common units of flow, such as cubic feet per second (ft^3^/s), by applying the appropriate units conversion. The catchment flow values were assigned to their respective stream reaches based on the unique identifier shared between the reaches and the associated catchments.

### Accumulation of Flow Downstream Through the Network

Flow delivered to the stream network from the catchments was accumulated in the downstream direction by using topological information associated with the MRB_E2RF1 reach network (Figure 2). The topology of the network defines the connections and flow directions of the stream segments. This allows any characteristic associated with the stream segments to be accumulated throughout the river network from the most upstream reaches (headwaters) to the most downstream reaches (coastal or inland terminal segments). In the case of accumulating flow throughout the reach network, the flow from all headwater reaches (HYDSEQ = 1 and HYDSEQ = 2 in Figure 2) is added to the flow of the next downstream river segment (identified as HYDSEQ = 3 in Figure 2). The correct downstream segments are determined by matching the TNODE of the upstream reach to the FNODE of the downstream reach. After the flow from all headwater reaches has been added to all the neighboring downstream reaches, the process is repeated for successively increasing HYDSEQ numbers until the terminal ends of the network are reached (Schwarz *et al.*, 2006).

### Removal of Local Bias in Estimated Flow

**FIGURE 5:**
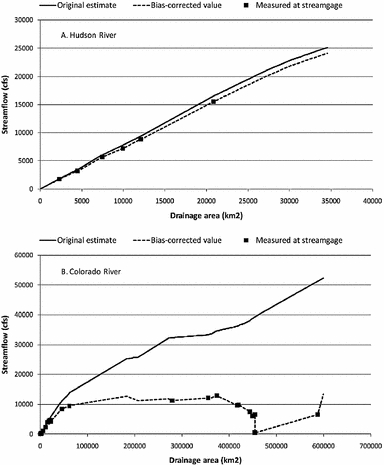
Estimated and Measured Daily Average Streamflow (water-year 2000) for River-Reach File 1 (RF1) Stream Reaches Along the Main-Stems of the (A) Hudson River and (B) Colorado River. The original flow estimate (sold line) is calculated by first intersecting the hydrologic unit runoff grid with MRB_E2RF1 catchments and then accumulating the flows downstream through the river network. The adjusted flow (dashed line) is computed by comparing the original flow estimates to flow values measured at streamgages, calculating the bias in the original flow estimate, and then interpolating the bias throughout the river network.

The accuracy of the accumulated estimated flow was evaluated by comparing measured and estimated flow values at streamgaging stations. One way to visualize the accuracy is by tracking measured and estimated flow along main-stem river corridors. Two examples of main-stem corridors, the Hudson River and the Colorado River, are shown in Figure 5. Agreement between the estimated (solid line in Figure 5A) and measured (black squares) flows is reasonable along the Hudson River from its headwaters to its downstream terminal end at New York Bay, although the original estimates are higher than the streamflow measured at streamgaging stations. In contrast, the measured (black squares) and estimated (solid line) flows diverge significantly along the Colorado River (Figure 5B), except in the headwaters. The poor performance of the model along the Colorado River occurs because the routing approach assumes that flow is strictly gaining (conservative accumulation) through the river network. In other words, all flow delivered to the network from the catchments accumulates with no losses as water flows downstream. This assumption is reasonable for the Hudson River but clearly not so for the Colorado River. In streams that naturally “lose” flow, the water-table elevation adjacent to the channel often is lower than the water surface of the river. Water then flows downward and laterally away from the stream channel, resulting in a net loss of streamflow; this “lost” water either recharges the groundwater system or evaporates. Water also commonly is lost from a stream reach due to withdrawals for irrigation, public supply, and other human water needs (Hancock, 2002; Prudic *et al.*, 2006; Rushton, 2007).

The local bias in accumulated flows was quantified by calculating the difference between the estimated (solid lines in Figure 5) and measured (black squares) flows at streamgaging stations and then interpolating the bias throughout the stream network using the associated topological information (Perry *et al.*, 2004). The HYDSEQ, FNODE, and TNODE values were used in a FORTRAN computer program to identify upstream and/or downstream gaging stations for each reach. The most similar upstream and downstream gaging stations were identified for each reach based on drainage-basin area.

If a streamgaging station was present upstream but not downstream of a stream segment, then the bias at ungaged segment *s* was computed as: 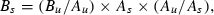
(1)where *B*_*s*_ is the bias at ungaged segment *s*, *B*_*u*_ is the bias at the upstream station, *A*_*u*_ is the drainage area of the upstream station, and *A*_*s*_ is the drainage area of ungaged segment *s*.

The bias correction method (Equation 1) is a heuristic approach in which the bias per unit area (*B*_*u*_/*A*_*u*_) at the upstream gaging station is multiplied by the drainage area of the ungaged segment (*A*_*s*_) and then weighted by the ratio of the drainage area of the upstream station (*A*_*u*_) to the drainage area of the ungaged segment. The ratio *A*_*u*_/*A*_*s*_ approaches a value of 1 when the drainage areas of the upstream gaging station and ungaged segment are very similar, and *A*_*u*_/*A*_*s*_ approaches a value of 0 when the drainage area of the upstream gaging station is much less than that of the ungaged segment. This method reflects the assumption that the estimated bias measured at the upstream gaging station should be given significant weight at ungaged segments near the station, but the bias measured at the upstream gaging station should have less weight when applied to ungaged segments far downstream from the gaged site. It should be noted that the bias correction is not constrained to conserve mass.

If there was a downstream gaging station but no upstream station, then the bias at ungaged segment *s* was computed as: 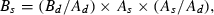
(2)where *B*_*s*_ is the bias at ungaged segment *s*, *B*_*d*_ is the bias at the downstream station, *A*_*d*_ is the drainage area of the downstream station, and *A*_*s*_ is the drainage area of ungaged segment *s*.

Equation (2) is similar in concept to Equation (1). The bias per unit area (*B*_*d*_/*A*_*d*_) at the downstream gaging station is multiplied by the drainage area of the ungaged segment (*A*_*s*_) and then weighted by the ratio of the drainage area of the ungaged segment to the drainage area of the downstream station (*A*_*d*_). The ratio *A*_*s*_/*A*_*d*_ approaches a value of 1 when the drainage areas of the downstream gaging station and ungaged segment are very similar, and *A*_*s*_/*A*_*d*_ approaches a value of 0 when the drainage area of the downstream gaging station is much greater than that of the ungaged segment.

If both upstream and downstream gaging stations are present, then the bias at ungaged segment *s* was computed as: 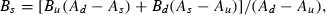
(3)where *B*_*s*_ is the bias at ungaged segment *s*, *B*_*u*_ is the bias at the upstream gage, *B*_*d*_ is the bias at the downstream gage, *A*_*d*_ is the drainage area of the downstream gage, *A*_*u*_ is the drainage area of the upstream gage, and *A*_*s*_ is the drainage area of ungaged segment *s*. Equation (3) interpolates the bias between two gages according to differences in drainage areas of the upstream gage, the downstream gage, and the ungaged segment. For example, when the drainage area of the ungaged segment is equal to the drainage area of the upstream or downstream gage, then the bias at the ungaged segment is equal to that of the upstream or downstream gage, respectively. When the ratio of the drainage area of the ungaged segment is exactly half that of the downstream gage and twice that of the upstream gage, then the bias measured at the upstream gage is given equal weight to that of the downstream gage.

Addition of the interpolated bias to the accumulated estimated flows throughout the river network generates bias-corrected flow estimates (the dashed lines in Figure 5) that exactly match the measured values at streamgaging stations. In the river reaches between streamgaging stations, the bias-corrected flow estimates produce a smooth pattern consistent with the streamflow values measured at the streamgaging stations. The difference between the original and adjusted flow estimates is most noticeable in arid-region river corridors, such as along the Colorado River, where losing stream reaches are common due to human activities and natural processes.

The entire procedure (estimation of runoff, intersection with catchments, accumulation of flows in the reach network, and removal of local bias) was applied to all stream reaches for each individual water year from 1971 to 2000 and then the average was computed for the entire 30-year period.

The ability to estimate mean-annual streamflow at ungaged reaches illustrates the benefits of using the river network as a component of the flow-estimation technique. The river network explicitly provides spatial connections between the ungaged reaches and the gaged streams. As represented by the bias-correction equations, knowledge of flow conditions upstream and downstream of ungaged reaches improves flow estimates compared to methods that do not incorporate network connectivity. These equations represent a type of spatial interpolation that is unique to river networks and cannot be approximated by simple Euclidean spatial methods. In a network-based interpolation, proximity between a streamgage and an ungaged reach is determined by upstream and downstream position within the network. In addition, similarity between the gaged and the ungaged reach is a function of the ratio of drainage areas, not linear distance.

## Summary

Regional-scale digital hydrologic networks used within the United States typically have been constructed from elevation points and hydrologic features collected by national mapping programs. The networks comprise hydrologically connected stream-reach segments depicting surface-water pathways and their associated drainage catchments. They can provide a consistent framework for descriptions and characterizations of aquatic and watershed processes controlling the supply, fate, and transport of constituents. Topological properties inherent to the networks provide the ability to simulate the movement of water and associated constituents. Collectively, these network characteristics are key components to hydrologic analysis and modeling. One such modeling application is SPARROW, a hybrid statistical approach that establishes relations between monitored contaminant flux, contaminant sources, aquatic transport processes, and the physical characteristics affecting transport. Explanatory properties associated with the network are evaluated to assess their significant contribution to supply and transport relative to the monitored flux.

Nutrient SPARROW models presented in this issue have been developed to address water-quality issues throughout the conterminous United States. Supporting these regional models, are two separate digital hydrologic networks derived from the 1:500,000 River Reach File and 1:100,000 NHD (medium-resolution, enhanced to create NHDPlus). RF1 networks have been enhanced and modified to support various applications of SPARROW modeling, both regionally and nationally. The current national geospatial dataset contains topological and stream characteristics necessary to execute a SPARROW model. These characteristics reside in a single table and include reach topology, hydrologic sequencing, reach identification, mean-annual streamflow, velocity, travel time, reach length, reservoir surface area, and stream-reach type. Delineated drainage areas for each reach provide the ability to spatially link watershed characteristics to the stream network. Because of a simplistic structure, the RF1-based network is a viable choice for a hydrologic network supporting applications like SPARROW. It provides an adequate representation of hydrologic features and pathways at a regional or national scale.

The need for accurate and updated stream characteristics prompted the use of the modified RF1 spatial framework to generate annual streamflow for each reach from 1971 to 2000. These updated flows were subsequently used in the regional SPARROW models presented in this issue. The equations used for the flow-estimation technique represent a type of spatial interpolation that is unique to river networks. Spatially explicit connections between ungaged and monitored locations provide knowledge of flow conditions upstream and downstream of ungaged reaches. In addition, similarities between the gaged reach and the ungaged reach are computed as a function of the ratio of associated drainage areas, not linear distance. This methodology was constructed such that it also could be used to improve the accuracy of the current estimated flows in more spatially detailed datasets like the NHDPlus.

NHDPlus is an application-ready product based on a 2005 snapshot of three core national databases: NHD, NED, and WBD. Regionally, NHDPlus provides significant spatial detail (1:100,000 medium-resolution) and a realistic representation of hydrologic pathways and numerous spatially referenced landscape and climatic characteristics. NHDPlus also contains appropriate topological and stream characteristics necessary to execute a SPARROW model. Additionally, an expanded capability of NHDPlus provides 30 m elevation-derived catchments and flow paths useful for more detailed spatial referencing and network generation. NHDPlus also benefits from a multi-agency coordination of NHD. Specific protocols for updating tools, features, and related information can potentially minimize any duplication of effort among stewards, incorporate corrections applied by SPARROW modelers, and provide a consistent dataset to the greater community.

NHDPlus was constructed to meet a variety of needs and applications. Therefore, the complexity of NHDPlus resides in the components that also are dependent on changing and evolving databases like NHD, NED, and WBD. NHDPlus also contains a vast number of records in which relations between surface-water flow paths and other associated characteristics are not as simplistic as in RF1. Many external tables contain numerous attributes related by common fields to the spatial locations of hydrologic features. Standardized tools or scripts could automate and simplify procedures for establishing these relations and extracting the data for a specific use in models like SPARROW.

As with many stream networks, the source information for mapping the hydrologic features of NHDPlus were based on cartographic interpretations. Mapping anomalies such as varying stream densities and isolated or unconnected reaches currently are being identified and corrected. One approach to improving inconsistencies in drainage density may be to remove streams in the smallest catchments.

Hydrologic networks developed from elevation data may provide more flexibility for transport models like SPARROW. Elevation-based flow-path networks allow for the option of processing environmental information at a grid-cell level as opposed to a catchment, reach, or watershed level. This may provide a more precise way to aggregate source and transport characteristics and take advantage of additional monitoring and environmental explanatory information at finer scales. Like NHDPlus, the flow-path networks can be improved using drainage-enforcement techniques and the existing vector-based hydrography. NHDPlus also provides the necessary features that can be used to construct and evaluate a cell-based network supporting SPARROW modeling. In addition, detailed elevation data are becoming more readily available for larger areas. It also is feasible to assemble networks at various adjustable scales based on streamflows estimated by the techniques described in this article.

## SUPPORTING INFORMATION

Additional supporting information can be found in the online version of this article and is intended to supply the reader with additional information on the development of hydrologic networks supporting regional nutrient SPARROW models and the NAWQA Program.

**Figure S1.** Schematic outline of the processes used to generate catchments for each MRB_E2RF1 reach.

Please note: Neither AWRA nor Wiley-Blackwell is responsible for the content or functionality of any supporting materials supplied by the authors. Any queries (other than missing material) should be directed to the corresponding author for the article.
