# Supplementary material for: Digital Hydrologic Networks Supporting Applications Related to Spatially Referenced Regression Modeling
Source: J Am Water Resour Assoc. 2011 Oct;47(5):916–32. doi: 10.1111/j.1752-1688.2011.00578.x (PMC3307631; doi:10.1111/j.1752-1688.2011.00578.x)

Digital Hydrologic NetworkS Supporting ApPLICATIONS RELATED TO Spatially Referenced Regression Modeling.

J.W. Brakebill^1^, D.M. Wolock^2^, and S.E. Terziotti^3^

### ^1^Geographer, Maryland-Delaware-D.C. Water Science Center, U.S. Geological Survey, 5522 Research Park Drive, Baltimore, MD, 21228USA; ^2^ Research Hydrologist, Kansas Water Science Center, U.S. Geological Survey, 4821 Quail Crest Place, Lawrence, KS, 66049; **^3^**IT Specialist, North Carolina Water Science Center, U.S. Geological Survey, 3916 Sunset Ridge Rd., Raleigh, NC, 27607 (E-MAIL/Brakebill; [jwbrakeb@usgs.gov](mailto:jwbrakeb@usgs.gov))

# Supplemental Information

Introduction

In support of the USGS NAWQA Program designed to assess the status of the nations water-quality, twelve (six nitrogen and six phosphorus) SPAtially Referenced Regression on Watershed attributes (SPARROW) models in six regions of the United States were recently developed to help describe the distribution, sources, and transport of nutrients in selected streams and rivers in the United States (Preston *et al*., 2009).

A stream reach network of surface-water pathways is the fundamental building block for the SPARROW modeling framework. Two principal geospatial datasets were used to support regional SPARROW modeling that provide the basic foundation for the reach networks. They include the RF1 and NHD geospatial datasets. RF1 has been edited and modified on numerous occasions in order to support regional and national SPARROW applications (Alexander *et al*., 1999; Nolan *et al*., 2002; Brakebill and Preston, 2003; Hoos *et al*., 2008). NHD has been modified in the New England area to support a regional nutrient SPARROW model (Moore *et al*., 2004). Similar enhancements to NHD have since been incorporated nationally into what is now known as NHDPlus (USEPA and USGS, 2005). MRB study units 2, 3, 4, 5, and 7 began with an enhanced version of a RF1 based stream-reach network (Nolan *et al*., 2002). MRB 1 in the Northeast used the more spatially detailed NHDPlus dataset.

A set of measured constituent loads in streams representing a wide variety of watershed conditions is a primary requirement for regional SPARROW model calibration. (Schwarz *et al*, 2006). Six regional nutrient datasets were compiled for use in each of the MRB study unit SPARROW models (Saad *et al*., 2011). Site location and water quality data were assembled and annual loads were computed for the period from 1970 to 2007 using methods described in Saad, 2011. Locations of each monitoring station were used to associate the site with the appropriate stream reach of the NHDPlus dataset (USEPA and USGS, 2005) and subsequently on an enhanced RF1 dataset (MRB_E2RF1). Over 3,500 water-quality monitoring sites were evaluated for referencing on the digital stream network. Although developed for SPARROW model applications, the load estimations and spatial referencing of the monitoring sites are suitable for other large-scale watershed studies and applications of various scales.

Catchment boundaries for each reach are used to summarize explanatory information used in the SPARROW models (Wieczorek and LaMotte, 2011a; 2011b). These basin characteristics are typically generated using GIS “overlay” and “combine” operations. Calculations usually include mean or accumulated values but also can include categorical values. Other catchment characteristics include local and total drainage area and the number of reservoir impoundments within a local catchment. Stream-reach characteristics and associated catchments used in the MRB 1 SPARROW model were derived from methods described in USEPA and USGS, 2005, and Johnston *et al*., 2009.

Spatial referencing

The ERF1_2 stream-reach dataset, based on RF1 (Nolan *et al*., 2002) is the base stream-reach network for nutrient SPARROW models developed in MRB’s 2, 3, 4, 5, and 7. After modifications, the resulting dataset supporting MRB SPARROW modeling for the above MRBs is referred to as MRB_E2RF1. This dataset can be found on <http://water.usgs.gov/GIS/metadata/usgswrd/XML/mrb_e2rf1.xml>.

Catchments delineated for each reach reside in a dataset called MRB_E2RF1WS and can be found on: <http://water.usgs.gov/GIS/metadata/usgswrd/XML/mrb_e2rf1ws.xml>.

Modifications and descriptions of MRB_E2RF1 and associated catchments (MRB_E2RF1WS) is the focus of the remainder of the supplemental information.

A new unique attribute, MRB_ID, was added to the MRB_E2RF1 stream-reach dataset to facilitate the association of additional water-quality monitoring stations to the data. To maintain a connection to earlier national SPARROW model applications, the default value for the attribute MRB_ID was set to the value of the previously used unique identification number, E2RF1##. The value of MRB_ID was modified as needed when new monitoring sites were associated with the network.

Six regional nutrient monitoring datasets (one for each MRB) were compiled by the MRBs from federal, state, local agency and university water-quality data. Each water-quality monitoring station was associated with an appropriate streamgaging station and evaluated for specific criteria suitable for estimating long-term nutrient loads (Saad *et al*., 2011). This included evaluating the water-quality period of record and the location and period of record for the appropriate streamgage at or near the site. All potential load sites were initially located on the NHDPlus stream network (to facilitate construction of planned SPARROW models that would use the NHDPlus network) and then matched to a corresponding location on the MRB_E2RF1 network. Many of the USGS streamgaging stations had been previously associated with NHD (Stewart *et al*., 2006), thereby simplifying the process.

A semi-automated process was used to locate monitoring sites on MRB_E2RF1 reaches. Each station was evaluated individually to verify if it had previously been associated to the network. The station identification number maintained in the stream-reach dataset and the newly created geospatial dataset of load estimation sites was used for this validation process. If the station had previously been associated with the MRB_E2RF1 stream network, then no further action was required. If not, a visual inspection of the station location, the hydrologic features in the NHDPlus dataset, and the MRB_E2RF1 dataset within a GIS was performed. This visual inspection also provided station name, stream name, and distance from the reach to the monitoring station. If the station was within 250 m of the reach and the stream and station name were determined to be consistent, then the station was associated with that reach. If the station was greater than 250 m from the reach, then manual methods were used to locate the station based on an evaluation of the stream and station names. In some instances, the location of the station was considered inaccurate, and the location of the station was adjusted. In other instances, it was determined that the station did not fall on a stream reach at this scale and, therefore, could not be used.

Associating a monitoring station with a reach required the placement of a new stream network node at the location of the monitoring station along the reach. This essentially “split” the existing reach into two separate reaches (Brakebill and Preston, 2003). Once a reach was split, the station identification number was assigned to the upstream reach. This upstream reach also received a new unique identification number (MRB_ID). Because the existing reach was split into two separate reaches, the travel time, which is a function of the reach length and stream velocity, was recalculated.

The process of locating monitoring stations on stream reaches identified additional issues that needed to be addressed. In some cases, water-quality stations were incorrectly located on the NHDPlus network. These sites were manually relocated on the appropriate reach. Some sites were co-located with other non-USGS agency sites but with different station identification numbers. In these situations, the station that measured water-quality information more suitable for load estimation was retained.

The spatial differences in scale between the NHDPlus and MRB_E2RF1 are noticeable (Figure 1, main article). In some cases, MRB_E2RF1 reaches were manually altered to better represent the stream channels at or near the location of the monitoring site. This insured that accurate catchments at or near the monitoring stations would be created. Manual editing involved moving the vertices of the stream reach to better match the stream locations mapped by NHDPlus.

Because each MRB study unit was at different phases of the project when the reach network was being compiled, hydrologic sequencing for each reach was calculated separately and independently within each MRB. The result is a sequencing number that lacks national consistency. However, for national SPARROW model applications, a new hydrologic sequence was later calculated that included all reaches nationally and is included in the dataset MRB_E2RF1.

Catchment Generation

After completing associations of monitoring stations to MRB_E2RF1, a drainage boundary (catchment) associated with each stream reach in the conterminous U.S. was delineated to create an area or zone for summarizing watershed characteristics. The term catchment refers to the local area that drains directly to a stream reach. The source data for the drainage area delineation was a 100-meter resolution elevation dataset modified and generalized from the 30-meter National Elevation Dataset (NED), (USGS 1999; Falcone, 2003). This data represents a snapshot of the NED from approximately 2001 (Falcone, 2003). The elevation data were forced to conform to the drainage patterns defined by the locations of the stream reaches. This was accomplished by insertion of a raster representation of the streams into the elevation data. This process, also referred to as “stream burning” (Saunders, 2000) uses a tool developed by Hellweger and Maidment (1997) to create an artificially low stream channel to ensure that the elevation surface would flow towards the stream segments. Depressions and sinks were removed from the elevation dataset and the stream network patterns were incorporated into the DEM, after which individual catchments were created for every uniquely identified stream reach.

The conterminous United States was divided into eight regions to expedite the catchment generation process. These regions coincided with MRB study unit boundaries (Preston *et al*., 2009). The following steps were used to create the catchments for each stream reach (Figure S1):

1. Three 100-meter resolution surface elevation grids representing eastern, central, and western sections of the United States were acquired (Falcone, 2003) and edited. These grids were based on the National Elevation Dataset (NED) (USGS, 1999). Elevation cells lacking valid elevation values were eliminated. The three elevation grids were merged into a national digital elevation model (DEM).
2. Shuttle Radar Topography Mission (SRTM) (Jarvis and all, 2008) data were used to delineate stream networks in areas of Canada and Mexico that contribute flow into the United States. These elevation derived stream networks were created to be consistent with the stream density of the MRB_E2RF1 data. The stream reaches were generated from 90-m flow direction raster data derived from the SRTM data which were conditioned by filling depressions in the surface and accumulating grid cells in the direction of streamflow – a threshold of 5000 accumulated cells constituted a stream reach. The stream network was thinned to match the reach network segments from the RF1 that pass through the United States / Canadian border and the United States / Mexican border.
3. The stream reaches representing foreign drainages were connected with the existing MRB_E2RF1 reaches to produce a continuous stream network. The reaches representing foreign drainages were populated with a unique identifier value not previously used in the United States.
4. The 100-meter surface elevation grid of the United States was merged with the SRTM based international elevation data to create one elevation surface. The entire reach network (MRB_E2RF1 plus foreign streams) was “burned” into the elevation surface so that drainage is enforced based on the stream network (Saunders, 2000).
5. A flow-direction raster dataset that identifies the flow patterns throughout the network was created.
6. A raster representation of the stream network with the unique identifier of each stream reach as the cell value was created using a 100-meter cell resolution.
7. Watershed catchments for each set of unique cell values from the raster representation of the stream network were created.
8. Catchment grids from the MRB study units were merged to form a national representation of catchments.

A large buffered overlap area (20,000 m) between MRB study units was required so that the automated data processing at hydrologic unit boundaries would analyze the flow direction values correctly. Editing still was needed to finalize the seamless dataset with catchments that shared reach end points. The buffer area of regional catchment layers was reduced by 10,000 meters to reduce edge effects, the raster layers were merged, and discrepancies between the merged layers were examined and edited to create a seamless catchment layer for the conterminous U.S.

Review and Revise Reach Catchments

Several quality control steps were applied to test the accuracy of the delineated stream reach catchment areas. Catchments that were not considered valid based on visual inspection were edited and corrected. In addition to visual checks against elevation data and known hydrologic basin divides, two major quality-control steps were taken: (1) checking that a catchment was generated for each stream reach, and (1) checking that the accumulated areas of catchments upstream of associated monitoring stations were within twenty percent of published drainage areas for the monitoring site.

To verify that each stream reach had a representative catchment, the database relations based on the unique identification numbers for each reach and catchment were used as a common field. Because each catchment was tagged with the unique identifier of the stream reach it drained, a direct relation between the two geospatial data layers exists. Unmatched records based on the identification numbers relation identified missing catchments. If a catchment was not created, it was typically because the stream-reach length was less than 100m (the cell resolution of the elevation data used to create the catchment). A stream reach shorter than 100m may not have had a catchment delineated because the function that creates a raster representation of the stream reaches assigned the elevation cell to the longer reach within the same elevation cell. For SPARROW models to operate, it is not imperative that each “small” stream reach have an associated catchment. As long as the stream reaches are topologically connected, the reach network will succeed in routing dependent and explanatory information. However, missing catchments will not contribute additional incremental loadings from the catchment, thus loosing explanatory power. This step simply maintains a clean one-to-one relation between stream reaches and the catchments that helps with record keeping when constructing explanatory files for model input.

The raster catchment layer was then manually edited to add a one cell representation of the stream reach. In some cases, legacy issues with the base stream-reach network (Nolan *et al*., 2002) caused the watershed functions to create erroneous catchment delineations. These situations included reaches starting and stopping at the same nodes, forming a loop, or reaches that were partially overlapping each other, i.e., occupying the same space but having different identification values. As legacy issues were discovered, overlapping arcs were deleted so that a clean network could be used for catchment creation.

To validate the accumulated areas upstream of the monitoring sites, the stream network was traced upstream from each site using the topological information in the network. The stream reaches that were upstream of a monitoring site were identified and the associated catchments were selected. The sum of the areas of the selected catchments was compared to the published drainage area value reported in either the USGS National Water Information System (NWIS) (USGS, 2008) or in a State or USEPA database. If the drainage area values differed by 20% or more, then the stream reach and associated catchments were further evaluated to determine the source of the discrepancy.

Generally, three reasons can account for a discrepancy in drainage area comparisons: the stream reaches were improperly registered or digitized so that there was a misalignment with the elevation data, causing the enforcement of drainage to be inaccurate (the most common source of discrepancy); the elevation data were inaccurate; or the published drainage area values were incorrect. Corrections were done to either the stream reach network, the elevation data, or to the drainage area value.

Corrections to the stream reach network were necessary when the stream reaches were misaligned with the elevation data. These situations occurred when mapped streams crossed ridges and major divides rather than follow the natural flow direction. In some cases these were obvious occurrences such as a straight line connecting two nodes, rather than following any topographic features. In many cases however, the streams needed to be compared to either digital raster graphics (DRG's - the digital representation of a 1:24,000-scale topographic map) or to more detailed stream networks (such as the 1:100,000-scale National Hydrography Dataset (NHD). Isolated networks were also reviewed and verified. Corrections to the stream reach network were done to correct the geography, but minimize the creation of new reaches.

Once reaches were corrected, two methods were used for correcting the catchments associated with the reaches. If major changes to the reach network were needed, then the stream reaches were burned into the elevation data again and new catchments were delineated. This occurred in MRB study areas 3 and 4. If only small areas were edited, then the raster representation of the catchments was edited manually to reflect the drainage area of the new stream paths. Manual edits included changing the value of cells to represent the correct catchment area. In the cases where the elevation data were inaccurate, manual edits to the catchments were necessary using DRG's as a background guide. The cases of miscoded drainage area values could be checked against other datasets and corrected manually.

Literature Cited

Alexander, R.B., J.W. Brakebill, R.E. Brew, and R.A. Smith, 1999. Enhanced River Reach File 1.2 (ERF1), U.S. Geological Survey Open-File Report 99-457 (scale 1:500,000). <http://water.usgs.gov/GIS/metadata/usgswrd/erf1.html>, *accessed* January 1999.

Brakebill, J.W., and S.D. Preston., 2003. A Hydrologic Network Supporting Spatially Referenced Regression Modeling in the Chesapeake Bay Watershed. Environmental Monitoring and Assessment Kluwer Academic Publishers 81: 73 – 84.

Falcone, James, 2003, National elevation data, resampled to 100m: Written communication, U.S. Geological Survey, Reston, VA.

Hellweger, F.L., and Maidment, D.R., 1997, AGREE-DEM surface rconditioning system: Austin, Texas, University of Texas. [*http://www.crwr.utexas.edu/gis/gishyd98/quality/agree/agree.htm*](http://www.crwr.utexas.edu/gis/gishyd98/quality/agree/agree.htm), *accessed* October, 2007.

Hoos, A.B., Terziotti, Silvia, McMahon, Gerard, Savvas, Katerina, Tighe, K.C., and Alkons-Wolinsky, Ruth, 2008, Data to support statistical modeling of instream nutrient load based on watershed attributes, southeastern United States, 2002: U.S. Geological Survey Open-File Report 2008–1163, 50 p.

Jarvis, A., H.I. Reuter, A. Nelson, E. Guevara, 2008, Hole-filled SRTM for the globe Version 4, available from the CGIAR-CSI SRTM 90m Database. [http://srtm.csi.cgiar.org](http://srtm.csi.cgiar.org/), *accessed* June, 2008.

Johnston, C.M., T.G. Dewald, T.R. Bondelid, B.B. Worstell, L.D. McKay, A.H. Rea, R.B. Moore, and J.L. Goodall, J.L, 2009. Evaluation of catchment delineation methods for the medium-resolution National Hydrography Dataset: U.S. Geological Survey Scientific Investigations Report 2009-5233, 88 p. <http://pubs.usgs.gov/sir/2009/5233/>, *accessed* January, 2010.

Moore, R.B., C.M Johnston, K. W. Robinson, and J.R. Deacon., 2004, Estimation of total nitrogen and phosphorus in New England streams using spatially referenced regression models: U.S. Geological Survey Scientific Investigations Report 2004–5012, 42 p.

Nolan, J.V., Brakebill, J.W., Alexander, R.B., and Schwarz, G.E., 2002, [*Enhanced River Reach File 2*](http://water.usgs.gov/lookup/getspatial?erf1_2), (U.S. Geological Survey Open-File Report 02-40, Reston, Virginia).

Preston, S.D., R.B. Alexander, M.D. Woodside, and P.A. Hamilton, 2009. [SPARROW MODELING--Enhancing Understanding of the Nation's Water Quality](http://pubs.usgs.gov/fs/2009/3019/), U.S. Geological Survey Fact Sheet 2009-3019, 6 p.

Saad, D.A., G.E. Schwarz, D. M. Robertson, N. L. Booth, 2011. A Multi-Agency Nutrient Dataset Used to Estimate Loads, Improve Monitoring Design, and Calibrate Regional Nutrient SPARROW Models. Journal of the American Water Resources Association, 10.1111/j.1752-1688.2011.00575.x

Saunders, W., 2000. Preparation of DEMs for use in envi­ronmental modeling analysis, *in* Maidment, D.R., and Djokic,  D., eds., 2000, Hydrologic and Hydraulic Modeling Support: Redlands, CA, ESRI Press, p. 29–51.

Schwarz, G.E., A.B. Hoos, R.B. Alexander, and R.A. Smith, 2006. The SPARROW Surface Water-Quality Model – Theory, Application, and User Documentation. U.S. Geological Survey Techniques and Methods, Book 6, Section B. Chapter 3, 248 p. <http://pubs.usgs.gov/tm/2006/tm6b3/>, *accessed* January 2007.

Stewart, D.W., A. H. Rea, and D.M. Wolock, 2006. USGS Streamgages Linked to the Medium Resolution NHD, U.S. Geological Survey Data Series DS-195. <http://water.usgs.gov/GIS/metadata/usgswrd/XML/streamgages.xml>, *accessed* June, 2008.

USEPA (U.S. Environmental Protection Agency) and USGS (U.S. Geological Survey), 2005. National Hydrography Dataset Plus (NHDPlus). <ftp://ftp.horizon-systems.com/NHDPlus/documentation/metadata.pdf>, *accessed* December, 2009.

USEPA (U.S. Environmental Protection Agency) and USGS (U.S. Geological Survey), 2009. National Hydrography Dataset Plus (NHDPlus) Users Guide, <ftp://ftp.horizon-systems.com/NHDPlus/documentation/NHDPLUS_UserGuide.pdf>, *accessed* December, 2009.

USGS (U.S. Geological Survey): 1999, National Elevation Data Base (NED): U.S. Geological Survey Fact Sheet 148-99. <http://mapping.usgs.gov/mac/isb/pubs/factsheets/fs14899.html>, *accessed* August, 2009.

USGS (U.S. Geological Survey): 2008. National Water Information System (NWIS): Web Interface. <http://waterdata.usgs.gov/nwis>, *accessed* May, 2008.

Wieczorek, M.E. and A.E. Lamotte, 2011a. Attributes for MRB_E2RF1 Catchments by Major River Basins in the Conterminous United States (U.S. Geological Survey Digital Data Series DS-491). <http://water.usgs.gov/nawqa/modeling/rf1attributes.html>, *accessed* February, 2011.

Wieczorek, M.E. and A.E. Lamotte, 2011b. Attributes for NHDPlus Catchments (Version 1.1) for the Conterminous United States (U.S. Geological Survey Digital Data Series DS-490).

<http://water.usgs.gov/nawqa/modeling/nhdplusattributes.html>, *accessed* February, 2011.


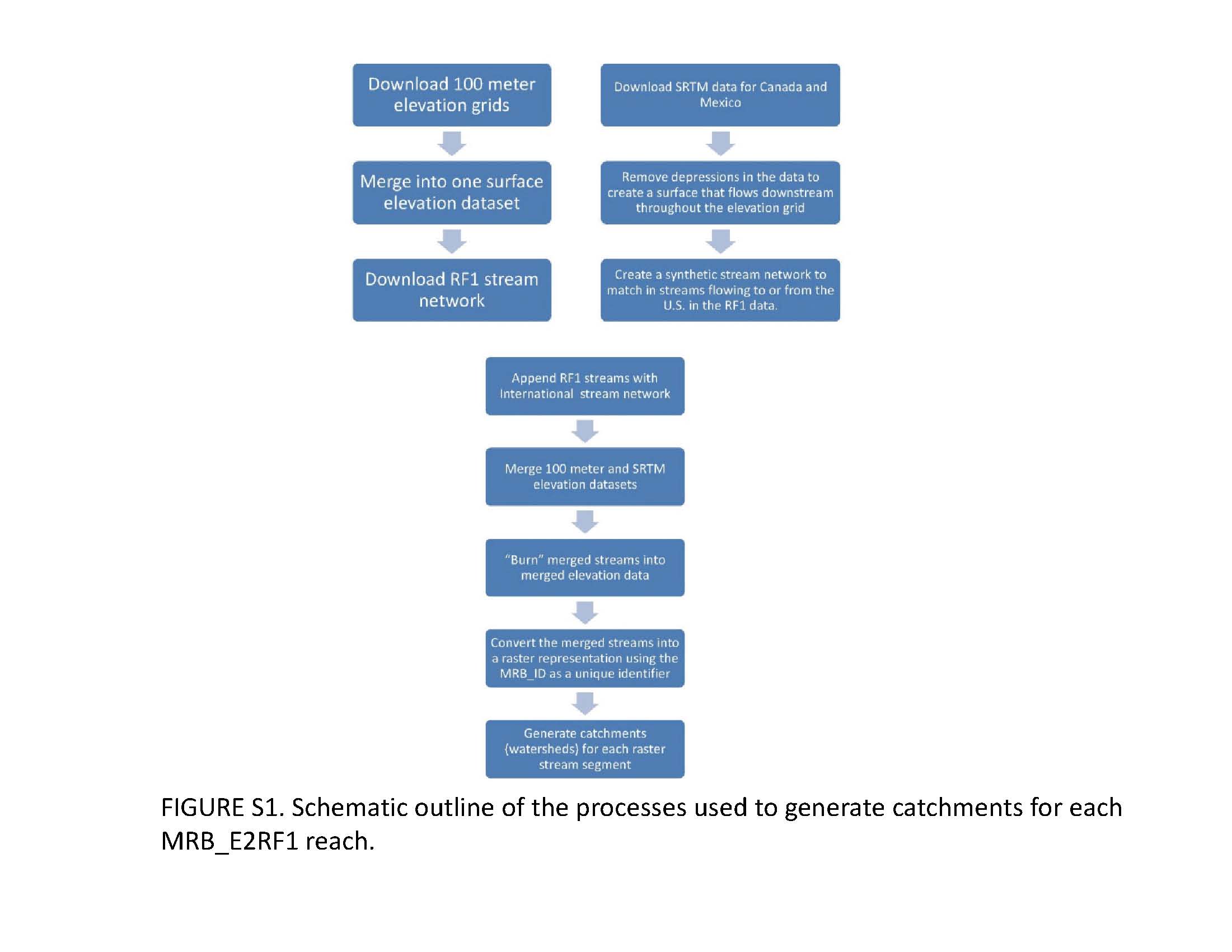

Supplement: Supplementary file 1 [file jawr0047-0916-SD1.doc]
